# Pharmacokinetics of Intramuscular and Intravenous Glycopyrrolate in Juvenile New Zealand White Rabbits

**DOI:** 10.1111/jvp.70061

**Published:** 2026-02-25

**Authors:** Jocelyn Marchiori, Andrea Sanchez, Kristen Messenger, Jennifer M. Reinhart, Hugues Beaufrère, Sonja Fonfara, Alexander Valverde, Yu Gu, Ron Johnson

**Affiliations:** ^1^ Department of Clinical Studies, Ontario Veterinary College University of Guelph Guelph Ontario Canada; ^2^ Department of Molecular Biomedical Sciences North Carolina State University Raleigh North Carolina USA; ^3^ Department of Veterinary Clinical Medicine, College of Veterinary Medicine University of Illinois Urbana‐Champaign Urbana Illinois USA; ^4^ Department of Medicine and Epidemiology UC Davis School of Veterinary Medicine Davis California USA; ^5^ Department of Biomedical Sciences, Ontario Veterinary College University of Guelph Guelph Ontario Canada

**Keywords:** anesthesia, bradycardia, glycopyrrolate, pharmacokinetics, rabbits

## Abstract

Glycopyrrolate is an alternative to atropine to treat bradyarrhythmias in anesthetized rabbits; however, there are no pharmacokinetic studies in the literature. Six female New Zealand White rabbits received glycopyrrolate 0.05 mg/kg intravenously (IV) in the cephalic vein or intramuscularly (IM) in a complete crossover design. Blood was collected via jugular catheter to determine glycopyrrolate concentrations at baseline, 3, 5, 10, 20, 30, 45, 60 min and 2, 4, 6, 8, 12 and 24 h post‐administration. Quantification of plasma glycopyrrolate was performed using liquid chromatography–tandem mass spectrometry. Non‐compartmental analysis showed fast clearance (104.03 ± 45.55 mL/kg/min), volume of distribution at steady state of 0.75 ± 0.54 L/kg and terminal half‐life of 2.49 ± 1.1 h after IV administration. Terminal half‐life was 3.34 ± 1.10 h, T_max_ was 0.07 ± 0.01 h, and C_max_ was 2.96 ± 2 ng/mL for the IM route. Absolute bioavailability for the IM route was 10.07% ± 3.39%, suggesting higher doses may be needed for this route of administration. This study reports the pharmacokinetic parameters of glycopyrrolate in rabbits after IV and IM administration and may help design future evidence‐based administration protocols in this species.

## 1 Introduction

Bradycardia is a common complication in rabbits under general anesthesia (Grint and Murison 2008; Barter and Epstein 2013; Gosliga and Barter 2015). Atropine is an anticholinergic often used to treat bradycardia in other species because of its quick onset of action and short duration of action (Fletcher et al. 2012; Lerche 2015). Current CPR guidelines in dogs and cat recommend the use of atropine in cases of cardiac arrest in the presence of asystole or pulseless electrical activity (PEA) associated with high vagal tone at a dose of 0.04 mg/kg (Hopper et al. 2024). However, atropine is not recommended in rabbits due to lack of efficacy and clinical effects (Olson et al. 1994). The reasons for its unreliability have been traditionally associated with the presence of circulating atropine plasma esterases, but even rabbits with no detectable levels of these enzymes show no clinical response to high doses of atropine, which has been attributed to rapid metabolism (Sawin and Glick 1943; Olson et al. 1994; Buckley et al. 2011; Lerche 2015; Flecknell 2016).

Glycopyrrolate is anecdotally used in rabbits instead of atropine. The rationale for this choice is that it is a synthetic anticholinergic with a longer duration of action than atropine (1 h vs. 30 min in dogs) and is less susceptible to hydrolytic degradation (Lerche 2015; Fletcher et al. 2012). Intravenous glycopyrrolate at 0.01 mg/kg caused similar increases in heart rate than 0.02 mg/kg of atropine 3 min after administration, showing similar onset of action in dogs (Richards et al. 1989). Despite this, specific dose recommendations for glycopyrrolate administration in rabbits are lacking. Doses recommended for dogs and cats (0.005–0.01 mg/kg) are often used in rabbits with inconsistent clinical results (Flecknell 2016; Fisher and Graham 2018). Results from one study suggest that much higher doses may be required to elicit a clinical response in rabbits (Olson et al. 1994); however, no pharmacokinetic data is available for this species. Based on the results of Olson et al. (1994), who found that 0.1 mg/kg of glycopyrrolate given intramuscularly prevented a decrease in heart rate in rabbits anesthestized with ketamine and xylazine, glycopyrrolate has the potential to have therapeutic effects in rabbits at higher doses.

We hypothesized that both intravenous (IV) and intramuscular (IM) administration of glycopyrrolate at 0.05 mg/kg will be well tolerated by healthy rabbits and that the IM route will show high systemic bioavailability and may be an alternative when IV access is not available. The objective was to measure plasma glycopyrrolate concentrations over 24 h following a single IV or IM dose of 0.05 mg/kg administered to healthy New Zealand White rabbits and report its pharmacokinetic parameters. Results from this study will help design an evidence‐based administration protocol for this drug in rabbits.

## 2 Methods

### 2.1 Animals

A total of 6 intact female research‐bred pathogen free New Zealand White rabbits aged approximately 3 months and weighing 2.77 ± 0.04 kg were used for this study (Charles River Laboratories Inc). The study was carried out in accordance with the guidelines of the Canadian Council on Animal Care and was approved by the Institutional Animal Care Committee at the University of Guelph (AUP #4697). Each rabbit was assessed and considered healthy for the experiment based on physical examination. Rabbits were boarded as small groups of 3–4 individuals in cage‐free housing for the duration of the study and were allowed to acclimate for 15 days before the study began. Water, clean bedding and litter boxes were always freely available. Enrichment was provided through areas to hide, climb and play. Rabbits were fed a balanced commercially available diet twice per day supplemented with hay, fresh fruits and vegetables and kept on a 12 h‐on, 12 h‐off light cycle. The same animals were later used for a dose–response in a separate study before going to adoptive homes.

### 2.2 Experimental Design

Rabbits had free access to water and food and were not fasted before isoflurane anesthesia for placement of a jugular catheter 24 h prior to the start of each experiment. Rabbits were mask‐induced with isoflurane (Fresenius Kabi, Canada Ltd) dialed to 5% and carried in 100% oxygen. Once the rabbits lost their righting reflex, isoflurane was maintained via face mask to effect to prevent movement during catheter placement. Skin was aseptically prepared with standard technique using 3 steps starting with a chlorhexidine gluconate solution 4% scrub, followed by isopropyl alcohol 70% swap and finished with chlorhexidine gluconate tinted solution 0.5%.

A cut down technique using a #15 scalpel blade and blunt dissection was used for direct catheterization of either jugular vein with a single lumen long‐term 20 Ga (2.6 Fr) × 12 cm (4.75 in) catheter with integrated extension set (MILA, Florence, KY). The skin around the catheter was closed with 4–0 PDS (Ethicon, INC, US) suture. The catheter was heparinized and bandaged in place with a layer of cotton wrap and a reinforced soft fabric collar on top that allowed securing the catheter in place with a Velcro strap. Following catheter placement, rabbits were recovered in individual cages.

A complete crossover design was selected to minimize the number of animals required. Animals were administered either IV glycopyrrolate (fast bolus within 1 s followed by saline flush) via a catheter placed in the cephalic vein (0.05 mg/kg, group IV; Glycopyrrolate injection USP 0.2 mg/mL, Sandoz Canada Inc., Quebec) or the same dose given IM in the epaxial lumbar musculature with a 24 G 1‐in. needle (group IM). Each rabbit received both treatments separated by a 15‐day washout period; a period > 10 elimination half‐lives based on reported data in horses and children (Rautakorpi et al. 1994; Rumpler et al. 2013). The treatment order (IV then IM vs. IM then IV) was randomly assigned using a block randomization with three rabbits per treatment group using the software random.org.

On the day of the experiment, fresh whole blood samples (1 mL) from the jugular catheter were collected into heparinized tubes, using a two‐stage sampling technique, before drug administration (time 0). Additional samples were collected at 3, 5, 10, 20, 30, 45, 60 min and 2, 4, 6, 8, 12 and 24 h post administration, for glycopyrrolate concentration determinations. Normal saline (Baxter) 1 mL was administered IV after every sample was collected. All samples were placed on ice immediately until plasma collection following centrifugation (3000 *x g* for 10 min) was completed (within 20 min of sampling time), then stored frozen at −80°C until they could be assayed.

A total of 14 mL of blood per rabbit was collected over 24 h in this study which does not exceed 10% of the total blood volume of each animal (total blood volume in healthy adult rabbits is 55–78 mL/kg; Richardson and Keeble 2014). The volume of blood sampled was also replaced by the same volume of crystalloid to minimize changes in hemodynamics.

Jugular catheters were removed after the 24‐h time point using mask‐induction of isoflurane as described above. Meloxicam, 0.5 mg/kg (Metacam 5 mg/mL Injection, Boehringer Ingelheim Animal Health Canada, Burlington, Ontario) was administered subcutaneously after the experiment was completed to provide analgesia. The contralateral jugular was used for the second phase of the study for each rabbit; no complications associated with jugular catheter placement or removal were seen other than mild skin irritation in 2 rabbits that resolved within 48 h.

### 2.3 Plasma Analysis of Glycopyrrolate

Analysis of plasma glycopyrrolate concentrations was performed using liquid chromatography–tandem mass spectrometry (LC–MS/MS) at the High‐Performance Liquid Chromatography and Mass Spectrometry Facility in the Department of Biomedical Sciences, University of Guelph.

A United States Pharmacopeia (USP) reference standard of glycopyrrolate was purchased from Sigma. Glycopyrrolate Iodide‐d3, as an internal standard, was purchased from Toronto Research Chemicals. Optima grade of formic acid, acetonitrile, and methanol were from Fisher Scientific; water was from Milli‐Q system (Millipore) and US Origin rabbit plasma was obtained from Innovative Research Inc. as matrix blank. Captiva Enhanced Matrix Removal (EMR)‐lipid plates from Agilent Technologies were used for plasma extraction.

Liquid chromatography analysis was performed on a Thermo Scientific Vanquish Flex Binary UHPLC system. Glycopyrrolate was separated on an ACQUITY Premier BEH C18 Column (1.7 μm, 2.1 mm × 50 mm, Waters, Ireland) connected with a Premier BEH C18 VanGuard FIT Cartridge (1.7 μm, 2.1 mm × 5 mm, Waters, Ireland). Auto sampler was kept at 4°C and column temperature was set at 35°C. The mobile phase consisted of A: ammonium formate buffer (10 mM) and B: acetonitrile with 0.1% formic acid (70:30 v/v) with a flow rate of 300 μL/min. A 2 μL sample was injected onto the column. Glycopyrrolate and its deuterated internal standard glycopyrrolate‐d3 were eluted at 1.19 min. The total LC run time was 4 min.

A Thermo Scientific Q Exactive Focus Orbitrap mass spectrometer equipped with a Thermo Scientific Ion Max source and a heated electrospray (HESI) source was used for MS analysis. Data were acquired in parallel‐reaction monitoring (PRM) positive ion mode. In this PRM mode, protonated [M]^+^ precursor ions were selected as m/z 318.21 for Glycopyrrolate, m/z 321.22 for glycopyrrolate‐d3.

The resulting MS/MS product ion spectrum was detected in the Orbitrap at a resolution of 17,500 (FWHM at m/z of 200) with AGC target set at 1e^5^. The ionization conditions were optimized using Tee infusion (10 mL/min) of glycopyrrolate (100 ng/mL) into LC flow (200 uL/min). The ion source parameters are optimized as: Spray voltage 3.5 kV, Capillary temp 300°C, S‐lens RF level 50.0, Aux gas heater temp 400°C. Stock solutions of glycopyrrolate (10 mg/mL) and glycopyrrolate‐d3 (1 mg/mL) were prepared by dissolving standards in methanol and stored at −80°C. Calibration standards, range of 5–5000 pg/mL were prepared on the day of analysis by spiking working solutions in blank rabbit plasma. To extract glycopyrrolate from rabbit plasma, a simple protein precipitation was carried out followed by the clean‐up of the extracts using Captiva EMR‐Lipid 96 well plate. Calibration Standards (5–5000 pg/mL) and quality controls (7.5 pg/mL and 4000 pg/mL) for glycopyrrolate were prepared on the day of analysis. Plasma samples with concentrations above the calibration curve range were diluted with blank rabbit plasma to obtain values within the calibration range.

The assay procedure was validated for specificity, selectivity, linearity, accuracy, intra‐ and inter‐day precision. Six different rabbit blank plasma samples were extracted and the background at the retention time of glycopyrrolate compared with the peak areas found in the lower limit of quantification (5 pg/mL) to determine the selectivity of the assay. No interfering peaks were observed in blank rabbit plasma at retention time 1.19 min corresponding to the drug glycopyrrolate. These clean backgrounds showed that the assay procedure was specific and selective with no interference from the sample matrix.

Calibration curves were prepared and assayed on 6 separate days. The 9‐point calibration curves were calculated using weighted (1/x) least squares linear regression. All curves were linear and reproducible with *R* ^2^ > 0.99 in the concentration range of 5–5000 pg/mL. The limit of quantitation (LOQ) was 5 pg/mL in this assay. LOQ was determined as the lowest concentrations with accuracy within 20% of nominal concentration and its precision was within 20% CV.

### 2.4 Pharmacokinetic Analysis

Given the small sample size, pharmacokinetic parameters are presented as geometric mean +/− standard deviation. Non‐compartmental analysis of the IV and IM data was performed using commercially available software (Phoenix WinNonlin 8.4, Certara L.P., Princeton, NJ). The terminal rate constant (λ_z_) was calculated from the slope of linear regression of time vs. log concentration of the terminal portion curve; the timepoints were included based on best fit (r^2^) of the regression line. Volumes of distribution were calculated as: V_ss_ = MRT * Cl, V_z_ = dose / (λ_z_) * AUC_0‐∞_, where V_ss_ is the volume of distribution at steady state, MRT is the mean residence time, Cl is clearance, V_z_ is the volume of distribution based on the terminal portion of the curve and AUC_0‐∞_ is the area under the curve extrapolated to infinity. For the purpose of these analyses, concentrations reported as below the limit of quantification (< 5 pg/mL) for the assay were set as 0 pg/mL (U.S. Food and Drug Administration 2018).

## 3 Results

**FIGURE 1:**
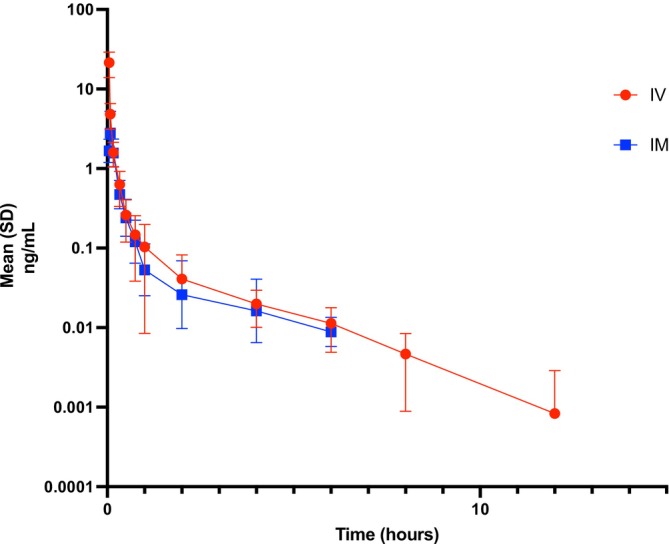
Semilogarithmic representation of mean plasma concentration versus time for 24 h after administration of 0.05 mg/kg of glycopyrrolate IV or IM given to healthy New Zealand White rabbits (*n* = 6).

**FIGURE 2:**
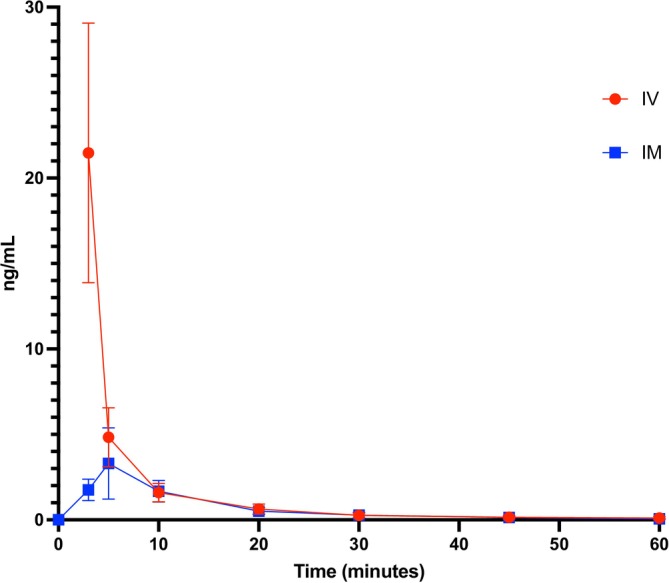
Numerical plasma concentration (ng/mL) versus time for the first hour after administration of 0.05 mg/kg of glycopyrrolate IV or IM given to healthy New Zealand White rabbits (*n* = 6).

All 6 rabbits completed both arms of the study. Non‐compartmental parameters for IV and IM glycopyrrolate administration are shown in Table 1. Figure 1 shows semilogarithmic plasma concentration of glycopyrrolate for IV and IM administration over 24 h. Due to rapid initial elimination of glycopyrrolate from plasma, numerical glycopyrrolate concentrations (ng/mL) over time for the first hour after administration are depicted in Figure 2. No side effects associated with glycopyrrolate administration were observed.

**TABLE 1:** Parameters from non‐compartmental pharmacokinetic analysis of glycopyrrolate in healthy New Zealand White rabbits (*n* = 6), after the administration of 0.05 mg kg^−1^ IV or IM. Data is expressed as geometric mean ± standard deviation.

Parameter	IV	IM
Cl (mL/kg/h)	6241.91 ± 2732.77	
Cl (mL/kg/min)	104.03 ± 45.55	
V_z_ (L/kg)	22.38 ± 11.86	
V_ss_ (L/kg)	0.75 ± 0.54	
MRT (h)	0.12 ± 0.04	1.19 ± 0.45
C_0_ (ng/mL)	248.94 ± 167.35	
T_1/2,λ_ (h)	2.49 ± 1.10	3.34 ± 1.10
T_max_ (h)		0.07 ± 0.01
C_max_ (ng/mL)		2.96 ± 2.00
AUC_0‐t_ (ng*h/mL)	7.98 ± 4.63	0.77 ± 0.31
AUC_0‐∞_ (ng*h/mL)	8.01 ± 4.63	0.81 ± 0.30
AUC_%extrap_ (%)	0.28 ± 0.21	3.46 ± 2.25
AUMC_0‐t_ (ng*h^2^/mL)	0.65 ± 0.49	0.53 ± 0.52
AUMC_0‐∞_ (ng*h^2^/mL)	0.96 ± 0.47	0.96 ± 0.50
λ_z_ (h^−1^)	0.28 ± 0.10	0.21 ± 0.07
F (%)		10.07 ± 3.39

Abbreviations: AUC, area under the plasma concentration curve; AUMC, the area under the moment curve; C_0_, the concentration immediately after IV administration; Cl, clearance; C_max_, the peak concentration; F, absolute bioavailability for the IM route; MRT, mean residence time; V_z_, volume of distribution by the area method; T_1/2,λ_, the terminal half‐life; T_max_, time to peak concentration; λ_z_, apparent terminal elimination rate constant.

## 4 Discussion

The objective of this pharmacokinetic study was to determine pharmacokinetic parameters after a single IV or IM dose of glycopyrrolate (0.05 mg/kg) in healthy female New Zealand White rabbits. The pharmacokinetics of glycopyrrolate in rabbits have not been previously reported. With the sample times used, the IM route showed very low bioavailability (≈10%) compared with the IV route, which may preclude IM administration of glycopyrrolate from clinical use in this species. In humans, the bioavailability of glycopyrrolate from the IM route was 66% (Ali‐Melkkila et al. 1989). Although the bioavailability of IM glycopyrrolate has not been documented in dogs or cats, in experimental studies, it appears effective at increasing heart rate when administered IM (Lemke 2001). The reasons for the low bioavailability found in this study are not known and may be multifactorial. As shown in Figure 2, plasma concentrations of glycopyrrolate decreased very quickly after IV administration and the peak plasma concentration found after IM administration was much lower. Two possible theories could explain this low bioavailability: first, the rate of elimination of glycopyrrolate in rabbits is faster than the rate of absorption, creating a flip‐flop like kinetic behavior.

This fast rate of elimination from plasma is supported by the high calculated C_0_ value after IV administration, relative to much lower IM C_max_. This AUC from C_0_ to first sample collected after IV administration comprised a large portion of the IV AUC, which in turn was much higher than the AUC after IM administration (and therefore produced low IM bioavailability). The second possibility is that the absorption of glycopyrrolate is lower than in other species due to differences in local perfusion or total surface area of muscle in contact with the injected solution.

This study reports pharmacokinetic results from a non‐compartmental model analysis. In adult human males that received a single bolus of glycopyrrolate (0.05 mg/kg, IV), a tri‐exponential decline with rapid and slow phase in distribution, followed by slow elimination phase was observed (Penttilä et al. 2001). Compartmental analysis from that study reported clearance of 16.8 mL/kg/min, with volume of distribution at steady state (Vd_ss_) of 0.37 ± 0.26 L/kg. Non‐compartmental analysis has been reported after IV glycopyrrolate administration in children at a dose of 0.05 mg/kg (Rautakorpi et al. 1994). Children were divided in 3 age groups of less than 1 year old, 1–3 years old and older than 3 years old; clearance was reported as 16.8–23.5 mL/kg/min, Vd_ss_ was 1.31–1.83 L/kg and T_1/2_ was 0.77–2.15 h. Rumpler et al. (2011) administered glycopyrrolate IV to Thoroughbred horses and found using non‐compartmental analysis that plasma clearance was 20.1 mL/kg/min, Vd_ss_ was 1.22 L/kg and T_1/2_ was 8.38 h. It appears that Vd_ss_ in rabbits (0.75 L/kg) is similar to values reported for children and horses while terminal half‐life values (2.49 h) after IV administration are much shorter than horse values and closer to children's values. Plasma clearance in horses is very similar to values reported for both adult and pediatric human patients, but results of this study suggest a much faster clearance in rabbits (104 mL/kg/min) compared with other species.

Non‐compartmental elimination half‐life was still longer than expected based on what is seen clinically in rabbits. It was also noted that there was a large disparity between volume of distribution at steady state and during the elimination phase (V_z_), both things may be related to method of analysis. Although results are not reported in this study, compartmental analysis was also performed for the IV phase with mono‐, bi‐ and triexponential models fit to the data. Best fit was selected using the Akaike information criterion. It was concluded that a 3‐compartment model best fitted the data, which has also been reported in humans and horses (Penttilä et al. 2001; Rumpler et al. 2013).

In drugs whose pharmacokinetics are best described by triexponential equations, a “deep compartment” is included in the conceptual model. This compartment is characterized by slow distribution back into the central compartment, which significantly slows elimination in the terminal portion of the time‐concentration curve (Riviere 1997). This compartment is characterized by slow distribution back into the central compartment, which significantly slows elimination in the terminal portion of the time‐concentration curve (Riviere 1997). Plasma concentrations of glycopyrrolate observed during terminal phase of the time‐concentration curve, which may represent elimination from the deep compartment, were very low and far below the suggested therapeutic threshold in other species since EC_50_ for glycopyrrolate in horses is 3.5 ng/mL and 2.46 ng/mL in humans (Penttilä et al. 2001; Rumpler et al. 2014). Thus, although the terminal half‐lives in the non‐compartmental analysis were longer than expected, these may not represent clinically relevant drug elimination from the body.

Total plasma clearance of glycopyrrolate, in humans and horses, is approximately equal to their hepatic blood flow (Davies and Morris 1993; Dyke et al. 1998), suggesting that renal elimination of glycopyrrolate is low (Rumpler et al. 2013). Hepatic blood flow in rabbits is approximately 60–65 mL/kg/min (Reeves et al. 1988; Demeure dit Latte et al. 1995), which is lower than the total clearance found in this study, indicating the possibility of extra hepatic clearance of glycopyrrolate. Rabbits as a species have a very fast metabolism and this may be in part why effective doses of some drugs in cats and dogs cannot be applied to rabbits and why they often require higher doses. This has been reported with multiple sympathomimetic drugs such as dopamine, norepinephrine or phenylephrine (Gosliga and Barter 2015; Uccello et al. 2020). Higher doses of these drugs are needed in rabbits to exert clinical effects when compared with other domestic species like dogs, cats or horses.

A possible limitation of this study is a small sample size, although samples of 5 or 6 animals are commonly used for descriptive PK studies in veterinary medicine and considered adequate since no statistical comparisons were made between groups. Another limitation is that rabbits used in this study were conscious, young, and healthy, which is not necessarily representative of the population experiencing bradyarrhythmias under general anesthesia or that are systemically ill or geriatric. Possible differences in PK parameters between sexes could also not be considered in this study since only intact females were used. Future studies should be done with glycopyrrolate administration in anesthetized rabbits, as this is the population that is more likely to experience peri‐anesthetic arrest and efficacious doses of glycopyrrolate have not been examined in this species.

In conclusion, glycopyrrolate administered IV or IM at 0.05 mg/kg was well tolerated by healthy rabbits with no observable side effects. Glycopyrrolate appears to have a fast clearance and large volume of distribution in healthy rabbits and very low availability when administered IM.

## Author Contributions

Jocelyn Marchiori: instrumentation and data acquisition, manuscript drafting. Andrea Sanchez: study design, instrumentation and data acquisition, data interpretation, manuscript drafting and revision. Alex Valverde: instrumentation and data interpretation, manuscript drafting and revision. Jennifer Reinhart: pharmacokinetic analysis, manuscript revision. Kristen Messenger: pharmacokinetic analysis, manuscript revision. Hugues Beaufrère: data interpretation, manuscript drafting and revision. Sonja Fonfara: data interpretation, manuscript drafting, and revision. Yu Gu: LC–MS/MS analysis and manuscript drafting. Ron Johnson: data interpretation, manuscript drafting and revision.

## Funding

This work was funded by a grant from Pet Trust of the Ontario Veterinary College.

## Ethics Statement

The authors confirm that the ethical policies of the journal, as noted on the journal's author guidelines page, have been adhered to during data collection. This study was also conducted in accordance with the guidelines of the Canadian Council on Animal Care and was approved by the Institutional Animal Care Committee at the University of Guelph, AUP #4697.

## Conflicts of Interest

The authors declare no conflicts of interest.

## Data Availability

The data that support the findings of this study are available on request from the corresponding author. The data are not publicly available due to privacy or ethical restrictions.
